# Intronic motif pairs cooperate across exons to promote pre-mRNA splicing

**DOI:** 10.1186/gb-2010-11-8-r84

**Published:** 2010-08-12

**Authors:** Shengdong Ke, Lawrence A Chasin

**Affiliations:** 1 Department of Biological Sciences, Columbia University, 1212 Amsterdam Ave, MC 2433, New York, NY 10027, USA

## Abstract

**Background:**

A very early step in splice site recognition is exon definition, a process that is as yet poorly understood. Communication between the two ends of an exon is thought to be required for this step. We report genome-wide evidence for exons being defined through the combinatorial activity of motifs located in flanking intronic regions.

**Results:**

Strongly co-occurring motifs were found to specifically reside in four intronic regions surrounding a large number of human exons. These paired motifs occur around constitutive and alternative exons but not pseudo exons. Most co-occurring motifs are limited to intronic regions within 100 nucleotides of the exon. They are preferentially associated with weaker exons. Their pairing is conserved in evolution and they exhibit a lower frequency of single nucleotide polymorphism when paired. Paired motifs display specificity with respect to distance from the exon borders and in constitutive versus alternative splicing. Many resemble binding sites for heterogeneous nuclear ribonucleoproteins. Specific pairs are associated with tissue-specific genes, the higher expression of which coincides with that of the pertinent RNA binding proteins. Tested pairs acted synergistically to enhance exon inclusion, and this enhancement was found to be exon-specific.

**Conclusions:**

The exon-flanking sequence pairs identified here by genomic analysis promote exon inclusion and may play a role in the exon definition step in pre-mRNA splicing. We propose a model in which multiple concerted interactions are required between exonic sequences and flanking intronic sequences to effect exon definition.

## Background

All pre-mRNA splicing reactions involve the removal of an intron from between two exons and so require the pairing of the splice sites at the two ends of the intron; such pairing can be considered as a mandatory 'intron definition' step in splicing. However, it is likely that the initial recognition of most splice sites also involves 'exon definition,' the identification of two splice sites across an exon. This idea was first put forth to explain the observation that appending a 5' splice site downstream of the second exon in a two-exon pre-mRNA molecule greatly enhances splicing of the upstream intron *in vitro *[1]. There has since been a wealth of genetic evidence supporting this idea: the common consequence of mutating one splice site in an internal exon is the skipping of the entire exon, leaving the wild-type splice site at the other end of the exon unused [2]. One can imagine exon definition as serving a quality control function, preventing splicing from occurring at an isolated splice site unless it results in the inclusion of a *bona fide *exon. Despite the wide acceptance of this idea, especially in metazoans where intron size is much greater than exon size, most biochemical investigations of splicing have focused on protein-protein interaction across introns, rather than on complexes that form across exons [3,4].

It is possible that spliceosomal components themselves mediate this concurrent recognition of splice sites [5,6]. For instance, a mutation in a 5' splice site that eliminates splicing can be suppressed by a mutation in the upstream 3' splice site that improves its agreement to the consensus [7]. However, given the surfeit of splicing regulatory motifs [8], it seems likely that exonic and/or intronic enhancers play a role in exon definition as well. Evolutionary changes that weaken a splice site can be compensated by changes in exonic splicing enhancer (ESE) or silencer (ESS) content and *vice versa *[9,10], implying that the exon in its entirety represents an evolutionary unit. Downstream intronic splicing enhancers (ISEs) show specificity for different classes of 5' splice site sequences [11] and could be contributing to exon definition. Specific and widespread combinations of motifs can also act negatively to promote exon skipping [12].

A simple first step in the end-to-end recognition of an exon could be the binding of proteins at the two ends of the exon that are capable of specifically interacting with each other. If there is a limited repertoire of such proteins, then their existence should be signaled by the occurrence of specific combinations of sequences that serve as binding sites for these putative exon definition factors. Such pair-wise combinations can act to promote the intron definition step in splicing. The binding of the same heterogeneous nuclear ribonucleoprotein (hnRNP) at the two ends of a long intron can promote splicing [13,14]. A computational search revealed motifs that co-occur at intron ends and such motif pairs were shown to promote intron removal [15].

Here we have sought evidence for *cis*-acting elements that act in combination at an earlier step in splicing, interacting from the two ends of an exon to mediate exon definition. Whereas most past computational searches for *cis-*acting splicing elements have focused on single motifs [16-18], here we have sought pairs of motifs that demonstrate an unusually strong tendency to co-occur across exons. We have limited ourselves to intronic motifs that are paired across exons for two reasons. First, there is increasing evidence that the intronic flanks of exons can play an important role in splice site recognition [15,19-21]. Second, a search for motif combination within protein-coding exons is complicated by the possibility of correlation due to the non-random association of protein motifs [22,23].

We found that more than 15% of exons harbor flanking motif pairs that are strongly associated with each other. These pairs are found around constitutive and alternative exons but not pseudo exons and their pairing is evolutionarily conserved. They are also associated most frequently with exons that appear relatively weak by other criteria. Specific pairs are also associated with tissue-specific genes. When tested in a heterologous context, these motif pairs were found to synergistically enhance exon inclusion. This enhancement proved to be context dependent, with specificity that was imparted by exonic sequences. Thus, the communication between exon ends may involve multiple interactions across the exon and its intronic flanks.

## Results and discussion

### Co-occurring motifs are found in the intronic flanks of exons

**Figure 1:**
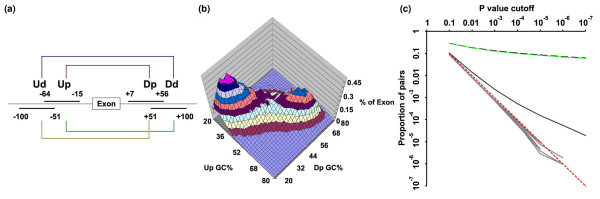
**Distribution of pentamer pairs around constitutive exons**. **(a) **Two intronic 50-nucleotide regions chosen on each side of an exon generate four possible pairings. Ud, upstream distal; Up, upstream proximal; Dp, downstream proximal; Dd, downstream distal. **(b) **The regions upstream and downstream of constitutive exons are highly correlated in GC content (Up and Dp shown here). The z-axis indicates the percent of exons whose combined 100-nucleotide flanks have the GC contents indicated on the x- and y-axes. **(c) ***P*-value distributions of constitutive exons and GC-balanced controls for the UpDp regions. The black line is the *P*-value distribution of constitutive exons with correction for GC content, the gray lines are the *P*-value distributions of ten GC balanced intron shuffled controls with correction for GC content, and the red dashed 45° line is the theoretical *P*-value distribution of the null hypothesis that the occurrences of upstream intronic motifs are independent of those of downstream intronic motifs. All *P*-value distributions of the ten controls matched the null hypothesis while the constitutive exons consistently generated substantially higher numbers of co-occurring motif pairs at different *P*-value cutoffs. The dashed black line is the *P*-value distribution for constitutive exons without correction for GC content. The dashed green line is the *P*-value distribution for the ten intron shuffled controls. These proportions without the correction are artifactually very high due to the high correlation of GC contents across limited genomic regions.

We extracted intronic regions upstream of the polypyrimidine tract of exons (upstream of -14 relative to the 3' splice site) and downstream of the consensus 5' splice site (downstream of +6 relative to the 5' splice site). We limited our search to 100-nucleotide intronic regions, as these have been seen to harbor distinctive motifs [15,19-21,24]. To examine regional specificity, we defined four 50-nucleotide stretches in which to search for co-occurring motifs: intronic regions from -100 to -51 nucleotides (Ud, upstream distal), from -64 to -15 nucleotides (Up, upstream proximal), from +7 to +56 nucleotides (Dp, downstream proximal), and from +51 to +100 nucleotides (Dd, downstream distal). Two intronic regions on each side of an exon generate four possible pairings: UpDp, UpDd, UdDp, and UdDd (Figure 1a). We chose pentamer pairs because this was the highest order k-mer for which our genome-wide study had sufficient statistical power. The sequence space for pairs of 6-mers is approximately 17 million. For 80,000 constitutive exons and 46 × 46 combinations of positions, an average of only 10 hits per pairing can be obtained, not enough to draw a statistically significant inference. Using 5-mers on the other hand means looking at only one million possible pairings and getting 170 hits per pairing, on average.

There are about one million pentamer combinations to consider when comparing two regions (4^5 ^× 4^5 ^= 4^10^). If we set the *P*-value cutoff at 1/4^10 ^(referred to hereafter as 10^-6^), we expect to see around one pentamer pair having a *P*-value smaller than this cutoff if pentamers in one intronic region are independent of those in the other region. Examining about 80,000 human constitutive exons, we found more than 60,000 pentamer pairs (approximately 6% of 4^10^) that passed this *P*-value cutoff. The top motif pairs detected all shared similar GC contents, being either GC-rich or AT-rich (shown for the UpDp region pairs in Figure S1a in Additional file 1 and Table S1 in Additional file 1). A GC content correlation between intronic regions flanking exons was expected due to the widespread occurrence of GC isochores in the human genome [25] and the exaggeration of this dichotomy in and around exons [20,26]. This GC content correlation is illustrated for the UpDp intron region pairing as an example in Figure 1b; the correlation coefficient (r) is 0.73. This strong correlation of the two intronic flanking regions is not observed for GA or GT content (r = -0.01 and 0.04, respectively; Figure S1b,c in Additional file 1).

To confirm our suspicion that these pairings were not specific, we performed a control experiment: the 50-nucleotide Up intronic region upstream of each exon was randomly exchanged with that of another exon having the same regional GC content; this procedure was then repeated for the downstream Dp region. This shuffling should greatly decrease the correlation if there were specific intronic pentamer pairs in the original pairings. No such decrease occurred and once again almost all of the pairs passing the 10^-6 ^*P*-value cutoff were either GC-rich or AT-rich. The *P*-value distribution of this shuffled control was quite close to that of the original constitutive exons, and both were substantially different from the null hypothesis model (Figure 1C).

**Figure 2:**
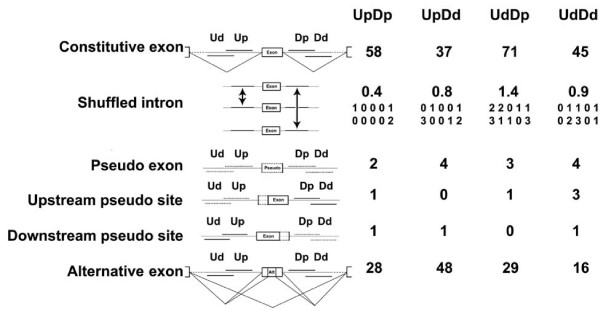
**Co-occurring motif pairs are found in intronic regions flanking exons**. Shuffled intron control: we randomly exchanged the 50-nucleotide intronic region of an exon with that of another exon if the two shared the same GC content. Both upstream and downstream intronic regions underwent this GC-balanced intron pairing randomization. This control destroyed the original upstream and downstream intronic region pairings while preserving the sequences inside the 50-nucleotide region. Each large numeral is the average of ten shuffles while small numerals show the individual results. Pseudo exons: these are defined as deep intron sequences of 50 to 250 nucleotides bounded by sites resembling 3' and 5' splice site consensuses but with no evidence of ever being spliced. Upstream pseudo sites: we searched the upstream intronic region of constitutive exons and found sequences with better 3' splice site scores than those of the real 3' splice site of the exon, but with no evidence of ever being used. Downstream pseudo sites: analogous to upstream pseudo sites. Alternative exons include cassette exons and those using alternative 3' or 5' splice sites.

To take this overriding GC content correlation into account in a search for specific pairings, we devised a method termed base bias corrected co-occurrence, or BBC-COOC. This algorithm greatly reduces the correlation due to GC content by restricting comparisons to exons with similar GC contents (see Materials and methods). A similar method was used by Friedman *et al. *[15] in a search for motifs co-occurring at the ends of introns. Applying this algorithm to UpDp, UpDd, UdDp, and UdDd intronic region pairings, we found 58, 37, 71, and 45 significantly correlated pentamer pairs, respectively, that passed the *P*-value cutoff of 10^-6 ^(Figure 2, row 1); the sum represents only 211 of the approximately one million possible pairs. We repeated the GC-balanced intron shuffle control described in the paragraph above for each of the four regional pairings. Ten repetitions of this control all generated only background numbers (approximately 1) of co-occurring motif pairs (Figure 2, row 2). Furthermore, *P*-value distributions of all ten control runs matched the null hypothesis while the constitutive exons consistently generated substantially higher numbers of co-occurring motif pairs at different *P*-value cutoffs (Figure 1C). The striking contrast between the constitutive exons and the controls confirmed the effectiveness of the BBC-COOC strategy in removing the GC content bias.

As an additional control, we asked whether the co-occurrence of pentamer pairs could also be found around other genomic sequences of a similar size. We examined pseudo exons [27], defined as deep intronic sequences of typical internal exon size (50 to 250 nucleotides) bounded by sequences resembling 3' and 5' splice sites, but which are never spliced. We applied the BBC-COOC algorithm to a large set (approximately 100,000) of nonredundant pseudo exons, using the same combinations of Ud, Up, Dp, Dd regions as for real exons. All four searches for correlations produced only numbers close to that expected for the null hypothesis (Figure 2, row 3). As a further control we examined the flanks of pseudo splice sites located upstream or downstream of real constitutive exons. That is, we searched the upstream intronic region of constitutive exons and found sequences with better 3' splice site scores than those of the exon and confirmed that these pseudo 3' splice sites were not used for splicing based on EST databases. Pseudo 5' splice sites were defined in the same way. We re-defined Ud, Up, Dp and Dd for these extended constitutive exons and checked the motif correlations of the four regional combinations with BBC-COOC. All four cases generated only background numbers of co-occurring motif pairs (Figure 2, rows 4 and 5). These results support the idea that the co-occurring motif pairs discovered in constitutive exon intronic flanks are involved in splicing and are not general features of the nonrandomness of the human genome. The discovery of particular significantly correlated intronic motif pairs located close to splice sites suggests that they may be working cooperatively across exons to promote exon definition and exon splicing. It may also be worth noting that the absence of co-occurring pairs around pseudo exons argues against such combinations being used to silence these false splice sites.

We next analyzed alternatively spliced exons using BBC-COOC and again found significantly co-occurring motifs. For three of the four regional classes, alternative exons gave rise to only about 40% of the number of motif pairs yielded by constitutive exons. This result might be attributable to the lower statistical power afforded by the smaller number of the former (approximately 35,000) compared to the latter (approximately 80,000). Interestingly, in the regional class UpDd, alternative exons yielded more co-occurring pairs than constitutive exons. This excess of alternative splicing motifs associated with a downstream distal region (more than +50 nucleotides) echoes the discovery of intronic elements regulating the alternative splicing of individual exons (for example, in the control of N-*src *splicing [28]) as well as with the global mapping of predicted Nova binding sites [29]. For most of the characterization of co-occurring motif pairs described below, we used the constitutive set to focus on exons with equally strong splicing.

Table S2 in Additional file 2 lists the co-occurring motif pairs found. The counts and *P*-values for all 1,048,576 pairs for each set of regions can be found at [30].

### Motif pairs occur close to splice sites

**Figure 3:**
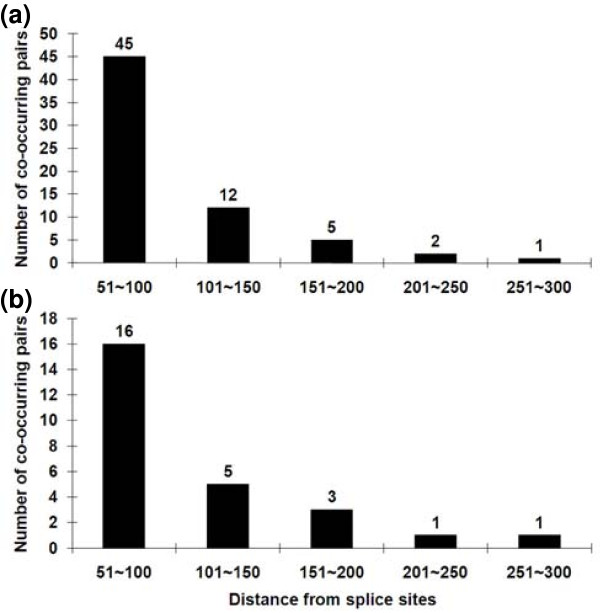
**Co-occurring motif pairs are enriched in intronic regions close to splice sites**. The BBC-COOC algorithm was used to search for significantly co-occurring motif pairs in symmetrically placed 50-nucleotide regions located at increasing distances from exon boundaries. The numbers of such pairs falls off sharply beyond 100 nucleotides and are reduced to background levels beyond 200 nucleotides. **(a) **Co-occurring motif pairs around constitutive exons. **(b) **Co-occurring motif pairs around alternative exons.

We determined the distance limits for regions harboring co-occurring motif pairs by extending the BBC-COOC analysis to pairs of 50-nucleotide stretches symmetrically spaced at 50-nucleotide intervals away from the borders of constitutive and of alternatively spliced exons. For both types of exons the frequency of co-occurring motif pairs dropped off sharply beyond 100 nucleotides from the exon borders but could still be detected out to about 200 nucleotides, although not further (Figure 3). These distance limits are similar to those found in computational searches for single motifs distinctive to the intronic flanks of exons [9,10,12] and are what might be expected for a role in exon definition [6,19-21,31].

### Co-occurring motif pairs exhibit regional specificity

**Figure 4:**
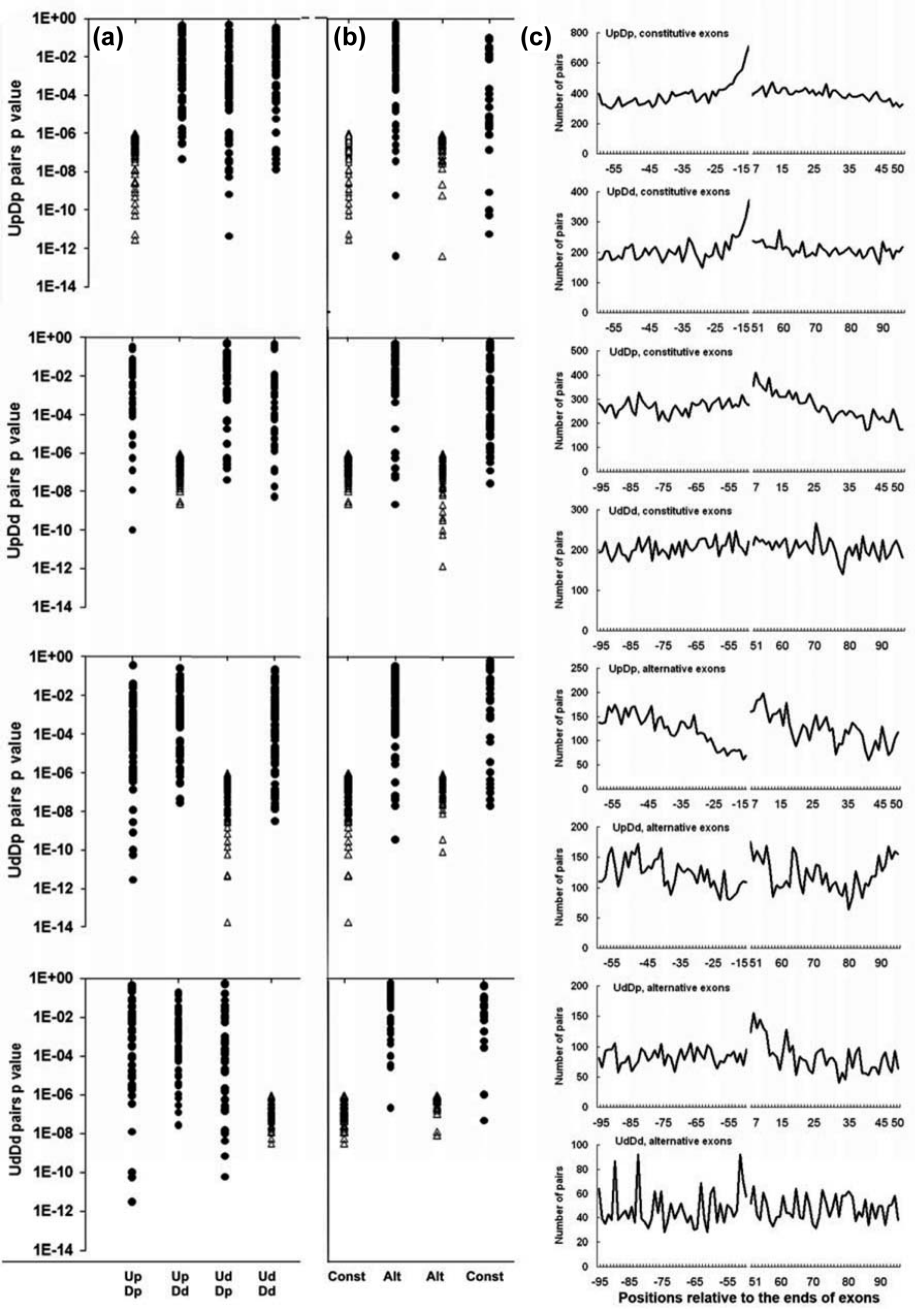
**Co-occurring motif pairs are specific for position and splicing efficiency**. **(a) **Regional specificity. Each row compares the *P*-values of the co-occurring pairs found in one regional class (open triangles; by definition less than 1/4^10 ^= approximately 10^-6^) with the *P*-values of those same motif pairs in the other three regional combinations (filled circles). Most of the co-occurring pairs were only significantly correlated for the regions in which they were discovered. **(b) **Constitutive exons versus alternative exons. Each row first compares the *P*-values of the co-occurring pairs found among constitutive exons (open triangles) with the *P*-values of those same motif pairs among alternative exons (closed circles), and then *vice versa*. **(c) **Positional distributions of co-occurring pairs around human constitutive and alternative exons. For each regional class the co-occurring motifs were enumerated at each nucleotide position in their respective 50-nucleotide regions, as indicated. Pentamers were counted on each side of an exon starting with the closest nucleotide. Approximately 120,000 constitutive exons and 70,000 alternative exons (including alternative cassette exons, alternative 3' splice site and alternative 5' splice site exons) were surveyed. D, downstream; U, upstream; p, proximal; d, distal.

Our consideration of two upstream and two downstream intronic regions created four pairwise combinations. We asked whether motif pairs that co-occurred in one combination of regions also co-occurred in another combination of regions. Motif pairs found in the UpDp combination all have *P*-values less than the *P*-value cutoff of 10^-6 ^by definition; very few of these motif pairs have *P*-values less than the *P*-value cutoff when examined in any of the other three regional combinations (Figure 4a).

We asked whether the lower number of motifs pairs passing the cutoff of *P *≤ 10^-6 ^in the other three regional combinations was due to a lower number of motifs and a consequent loss of statistical power. Such was not the case, as the expected number of motifs pairs (based on the number of individual motifs) was comparable in almost all cases; for 98% of the co-occurring pairs, the lowest number of expected pairs (based on the null hypothesis) was within a factor of two of that for the defining region (UpDp in this case). The same was true for the other three regional combinations shown in Figure 4a.

**Figure 5:**
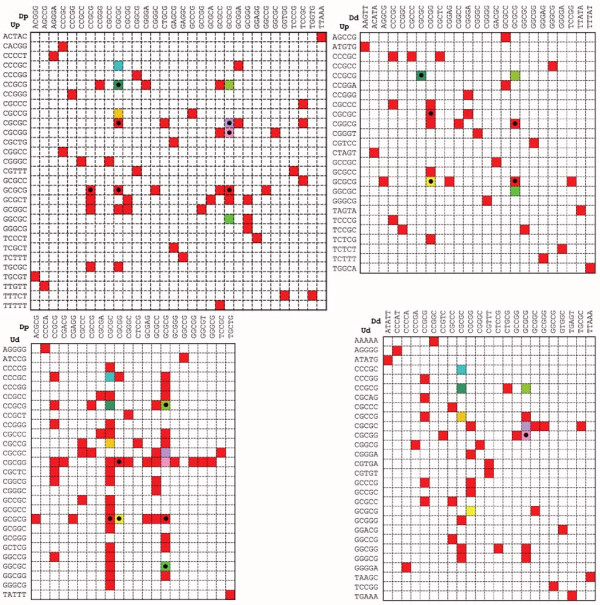
**Regional specificities and commonalities among co-occurring pairs**. Colored boxes define co-occurring pairs for each regional class. A red box indicates a sequence pair that is unique to a pair of regions, while other colors, all unique, indicate sequence pairs that are common to at least one other pair of regions. A black dot inside a colored box indicates a pair that is common to both constitutive and alternative exons.

If these motifs are cooperating to enhance splicing, then this cooperation may be quite sensitive to the distance between a motif and its nearest splice site. For example, motifs A and B may be able to cooperate to enhance splicing of an exon between them, but if motif B is moved 50 nucleotides closer to the splice site, this pair is no longer effective. Such context dependence has previously been seen for exonic splicing enhancers [18] and represents a major problem in deciphering the rules governing the regulation of splicing. The regional specificities of all individual co-occurring pairs are presented in Figure 5.

### Motif pairs around alternative and constitutive exons differ

In the same way, we asked to what extent motif pairs discovered around constitutive exons overlapped with those found around alternative exons. Here again we saw specificity: most of the pairs from constitutive exons that passed the 10^-6 ^cutoff were not among those that passed the cutoff from alternative exons and *vice versa *(Figure 4b). Because the cutoff is quite stringent, this result does not necessarily mean that the constitutive motif pairs are not found around alternative exons. But it could be interpreted to mean that alternative exons make greater use of special motif pairs. An interesting possibility is that the motif pairs found around alternative exons are actually acting negatively to promote alternative exon skipping. We explore this idea further below. Alternatively, the distinction may be secondary to tissue specificity, which is likely to be higher among alternatively spliced exons. The idea that the genes that harbor these constitutive exons are confined to just a few functional classes was ruled out by the observation that they comprise a very wide variety of Gene Ontology classes (data not shown).

### Motif pairs are conserved in evolution

**Figure 6:**
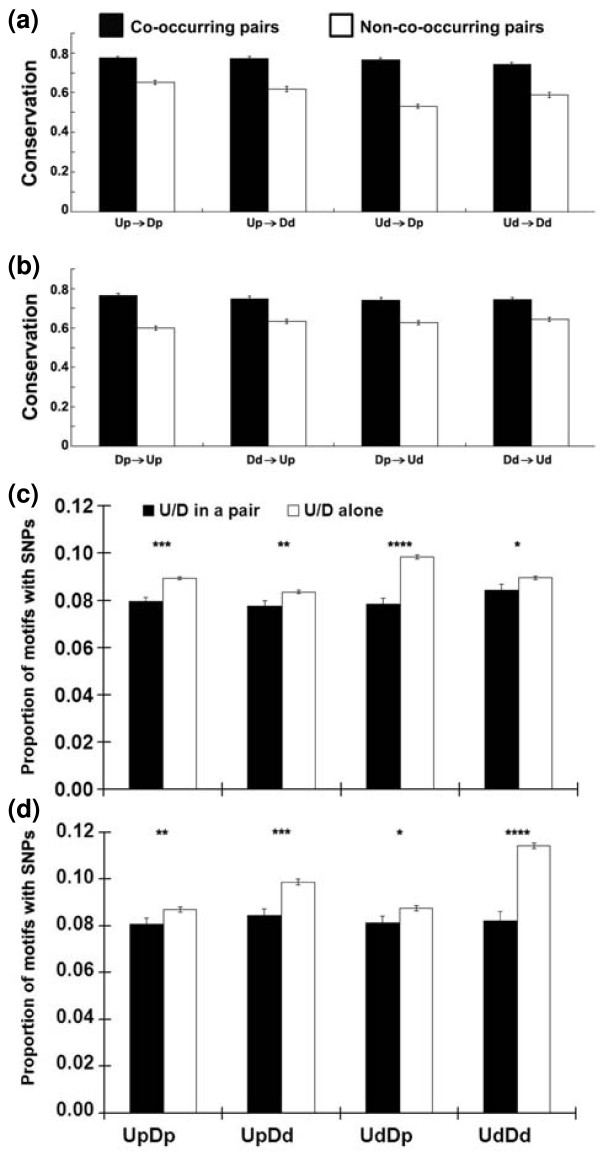
**Co-occurring pairs are conserved in evolution**. **(a) **Conservation of motif pairing in human and macaque. Conservation is defined as the proportion of orthologous constitutive exon pairs in which the upstream motif of a pair has been conserved given the conservation of a downstream motif (filled bars). The control (open bars) scored the conservation of non-co-occurring motif pairs (see text). **(b) **As (a), but in the other direction, scoring the conservation of the downstream motif given the conservation of the upstream motif. **(c) **Lower SNP density in intronic motifs of co-occurring pairs around constitutive exons. **(d) **Lower SNP density in intronic motifs of co-occurring pairs around alternative exons. The proportions of motifs containing SNPs were examined for the same set of motifs either when part of a co-occurring pair or when alone. Error bars are the standard error of the mean. *P < 0.05; **P < 0.01; ***P < 10^-7^; ****P < 10^-13^.

If co-occurring motif pairs interact across exons to promote splicing, then their pairing should be evolutionarily conserved. We addressed this question by comparing human and macaque sequences. For each of the four regional classes, we identified human constitutive exons that harbor co-occurring pairs and then collected the macaque orthologs of those exons [10]. Conservation of pairing was calculated as follows. If the region downstream of the macaque exon contained the downstream pentamer of the human co-occurring pair, then it was examined for the presence of the upstream pentamer in the corresponding upstream region. If the partner motif was found upstream, then the pairing was deemed conserved. We define co-occurrence conservation as the proportion of such successes. To provide a background for comparison, for each co-occurring pair, we chose a hexamer of the same base composition as the downstream partner but that did not significantly co-occur with the upstream partner (see Materials and methods). These calculations were then repeated for the conservation of the downstream partner given the conservation of the upstream partner. Starting with either the downstream or the upstream motif yielded the same result (Figure 6a,b): the conservation of pairing between co-occurring pairs (approximately 0.75) was significantly greater than the conservation of pairing when one partner was from a non-co-occurring pair (approximately 0.60, *P *< 10^-40^). The fact that the pairing of these motifs has been conserved in primate evolution supports the idea that they are functional, perhaps working in concert to promote exon splicing through exon definition.

### Co-occurring pairs have a lower SNP density

The co-occurring pair hypothesis predicts that mutations that occur in these motifs should have a higher likelihood of disrupting exon splicing than those that occur in the same motifs when they are alone. Therefore, the former would be more likely to be eliminated by purifying selection. Thus, the motifs of co-occurring pairs should have a lower SNP density. Consistent with this prediction, for all four regional classes the SNP density was significantly lower when the motifs were paired than when they were unpaired for both human constitutive exons and alternative exons (Figure 6c,d). This observation suggests that motifs of co-occurring pairs have been subject to purifying selection as pairs in recent human evolution and reinforces the conclusion from the human-macaque comparison. SNPs that disrupt a co-occurring pair could result in decreased exon inclusion, a lower level of the protein product and a mutant phenotype. In this way they may provide a class of functional markers for the identification of quantitative traits affecting human phenotypes, including disease associations.

### Motif pairs are associated with weaker exons

If intronic co-occurring pairs act to promote splicing, then they might be expected to contribute more frequently to exons that are otherwise relatively deficient in splicing signals. We compared all constitutive exons that contain co-occurring motif pairs of a particular class (that is, UpDp, UpDd, and so on) to the constitutive exons of a set that did not contain such pairs. The exons of the second set were exactly matched to the first set in the GC content of the relevant paired intronic regions so as to minimize the influence of base composition on any correlations seen. For instance, regions high in GC content will tend to be associated with splice sites that are high in GC content [32], which in turn are associated with poorer splice site consensus scores.

**Figure 7:**
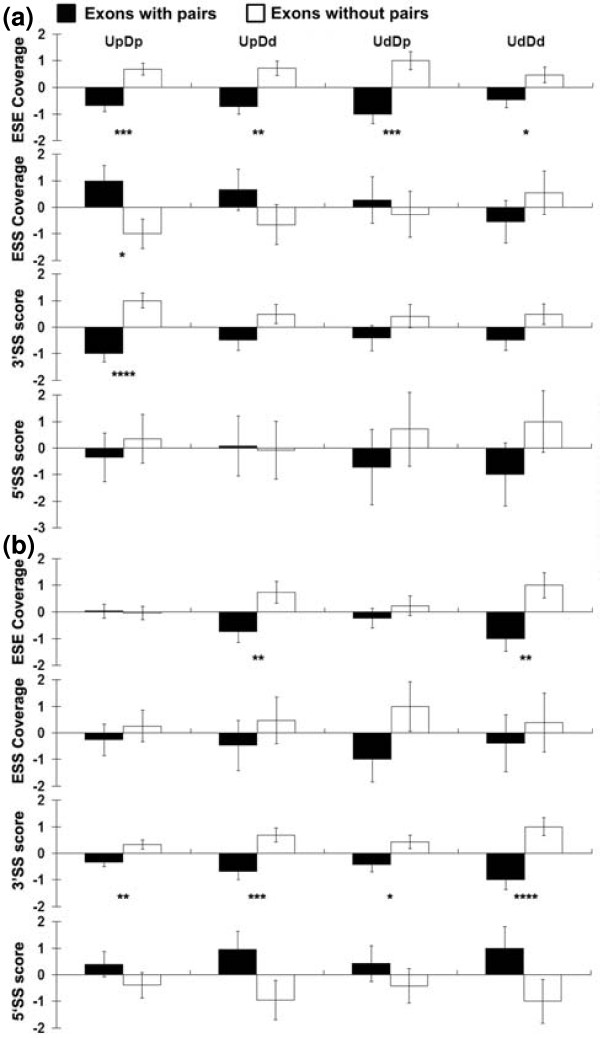
**Co-occurring pairs are associated with weaker exons**. **(a) **Two sets of constitutive exons were compared, one with co-occurring pairs in the indicated region and one without such pairs. The two sets were matched for GC content in the pertinent regions. ESE and ESS coverage refers to the proportion of exonic nucleotides that reside in a composite set of ESE and ESS hexamers [10]. 5' and 3' splice site scores are based on the method of Shapiro and Senapathy [50]. For each comparison the mean of the two exon sets was subtracted from all values to create a mean of zero and the maximum difference between the values of the two exon sets and this mean was set to 1; all other values were adjusted accordingly. All four UpDp, UpDd, UdDp, and UdDd combinations were treated separately. Error bars are the standard error of the mean. Asterisks below the bars indicate *P*-values: *P < 0.05; **P < 0.001; ***P < 0.0001; ****P < 0.00001. SS, splice site. The range of actual values across all four regional comparisons were: ESE coverage, 0.439 to 0.467; ESS coverage, 0.109 to 0.125; 3' splice site scores, 74.295 to 75.514; 5' splice site scores, 81.519 to 82.124. **(b) **As (a), but two sets of alternatively spliced exons were compared, one with co-occurring pairs in the indicated region and one without such pairs. The range of actual values across all four regional comparisons were: ESE coverage, 0.393 to 0.432; ESS coverage, 0.092 to 0.109; 3' splice site scores, 68.676 to 71.780; 5' splice site scores, 78.914 to 80.087.

Co-occurring motifs tended to have lower ESE coverage, higher ESS coverage and poorer 3' splice site scores compared to exons without co-occurring motifs (asterisked results in Figure 7a). These results support the idea that co-occurring pairs are contributing to splicing by compensating for a lack of strong splicing signals. That the association of higher ESS coverage with co-occurring pairs is not as strong as that of lower ESE coverage may be due to our inadequate definition of ESS sequences. Alternatively, intronic sequences acting in exon definition may be unable to compensate for the negative effects of exonic silencers.

Motif pairs associated with alternatively spliced exons might not have shown a correlation with weak exons if many motif pairs were acting to help silence rather than enhance splicing. However, the statistically significant results in the case of alternatively spliced exons also showed an association with weaker exons (Figure 7b), consistent with motif pairs enhancement of splicing for alternative as well as constitutive exons.

### Sequence characteristics of motif pairs

Most of the co-occurring pentamers are GC-rich (Figure 5; Table S2 in Additional file 2) and approximately 90% contain at least one CpG dinucleotide. This high CpG content is notable in light of the low general abundance of CpG in introns due to the mutational vulnerability of the oft-methylated C. Somewhat less than half of exons with co-occurring pairs harbor these CpG-containing motifs (41%). We considered the possibility that the high incidence of CpG dinucleotides in co-occurring pairs might be an artifact caused by internal exons that are located close to the 5' ends of transcripts. The transcription of most human genes is driven by CpG islands that lie upstream of the transcription start site, but that often extend several kilobases beyond it. If so, then pseudo exons should be subject to the same bias, as many of them would also be located near the 5' ends of genes, especially since first introns tend to be long [33] and would therefore be major contributors to the pseudo exon pool. The absence of co-occurring pairs from around pseudo exons (Figure 2) argues strongly against the possibility that these co-occurring pairs arise from CpG island transcription signals rather than from splicing signals. It should be noted that CpG-rich motifs are characteristic of the binding site of RBM4, a multifunctional RNA binding protein [34].

Despite the high GC content of most of these pentamers and their attendant sequence simplification, we saw no evidence for complementarity among them; perfectly complementary pairs appear at a frequency (7/211) no greater than that seen among random pentamers with the same overall base composition (for example, 10/211). Thus, secondary structure does not seem to be playing a role in the selection of these motif pairs.

### Comparison with previously generated intronic motifs

If the intronic motifs discovered here function to promote splicing, they may overlap with previously reported motifs computationally predicted to do the same. We compared the 38 unique downstream motifs from the constitutive and alternative UpDp classes with pentamers located in downstream intronic flanks that were predicted to be ISEs based on their relative abundance and/or evolutionary conservation [19-21,24]. There was little overlap among the ISEs (Table S3 in Additional file 1), perhaps because the co-occurring motifs are distinctive in their pairing rather than their individual relative abundances or conservation.

### Genomic distribution of motif pairs

The co-occurring motif pairs are abundant: overall, 17% of internal constitutive exons have co-occurring motif pairs in their intronic flank regions. The proximal UpDp combination yielded the greatest number of co-occurring pairs, but all combinations were substantially represented: UpDp, 7.6%; UpDd, 5.0%; UdDp, 3.5%; UdDd, 4.6% (these numbers add up to more than 17% because many exons have more than one class of pairs). Because we set a stringent *P*-value threshold for detecting these co-occurring pairs, the actual proportion of human constitutive exons with functioning co-occurring pairs may be much higher. This abundance would allow co-occurring motif pairs to play a role in the splicing of many human constitutive exons. For constitutive exons, motif pairs that originate from proximal regions tend to be clustered at the proximal end of the 50-nucleotide region (closer to the splice site); on the contrary, motifs from distal regions are spread throughout the distal region (Figure 4c). Interestingly, the clustering close to the 3' splice site is not seen among alternative exon motifs. Although the Up region spans the usual position of branch points, none of the Up motifs resembles that consensus (Figure 5; Table S2 in Additional file 2).

### Many co-occurring motifs resemble hnRNP binding sites

Many of the motifs in the co-occurring pairs resemble the binding sites of hnRNPs or other RNA binding proteins, including hnRNPs A1/A2, C, D, F/H,G, I (PTB), K, L, M, and 9G8 (Table S2 in Additional file 2); more than 30% of the individual motifs fall into this category. Almost all of these RNA binding site motifs are more characteristic of introns than of exons. While hnRNPs have been most often associated with splicing silencing, many of those examples involve binding within exons, and there are many other examples in which hnRNPs play a positive role in splicing from positions outside the exon [34]. The position of such binding sites relative to the exon can play a determining role in their mode of action, as exemplified by Nova sites, which are generally inhibitory downstream of exons but stimulatory upstream [29]. Computationally defined [16] or experimentally selected [35] exonic silencer sequences are enriched in the intronic flanks surrounding splice sites, where they may aid in accurate splicing by silencing nearby pseudo sites [16,36]. Chabot and colleagues have shown that two hnRNP A1 molecules can promote intron definition by binding to the two ends of an intron, with the idea that the interacting proteins bring those ends together [14]. It is tempting to speculate that hnRNPs may function in an analogous manner to bring the two ends of an exon together to effect exon definition.

### Genes containing exons with motif pairs show tissue-specific bias

Transcripts subject to tissue-specific alternative splicing often contain intronic regulatory sequences corresponding to the binding sites of splicing factors that are preferentially expressed in the corresponding tissues, such as Nova in brain [37] and Fox in muscle and brain [38,39]. Tissue-specific differences in the levels of ubiquitously expressed factors such as serine/arginine-rich proteins (SR proteins) and hnRNPs [40,41] (see Figure S3 in Additional file 1 for examples) can also play a role in tissue-specific alternative splicing [3,42]. Because many motifs of co-occurring pairs resemble binding sites of hnRNP proteins, we asked whether genes containing exons with those motif pairs would demonstrate tissue-specific biases.

**Figure 8:**
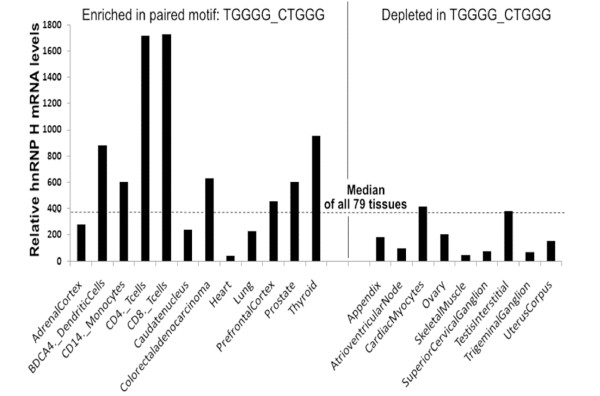
**Motifs resembling hnRNP binding sites correlate with tissue-specific preferential expression of the hnRNP mRNA**. Gene expression data were obtained from Su *et al. *[43]. Both 5-mers in the co-occurring pair TGGGG/CTGGG resemble the binding site of hnRNP H. *P *< 0.01, binomial test.

For this study we used a published survey of gene expression in 79 human tissues and cell lines [43] (see Materials and methods) and examined alternative exons containing co-occurring motif pairs significantly associated with alternative exons. We found that genes that have alternative exons with the motif pair TGGGG:CTGGG (both motifs resemble hnRNP H binding sites) in the UpDp intronic regions were significantly enriched among genes preferentially expressed in prefrontal cortex, thyroid and many immune tissues (BDCA4^+ ^dendritic cells, CD14^+ ^monocytes, CD4^+ ^T cells, and so on), and significantly depleted in appendix, superior cervical ganglion and skeletal muscle. Interestingly, this pattern correlated (*P *< 0.01, binomial test) with the tissue-specific expression levels of hnRNP H (Figure 8; Table S4 in Additional file 3). Genes that contain alternative exons having the motif pair CCCGG (hnRNP K binding site):TTTTT (hnRNP C binding site) in UdDd intronic regions exhibited significant enrichment in BDCA4^+ ^dendritic cells, CD4^+^T cells, CD8^+ ^T cells, hypothalamus and prefrontal cortex and were depleted in cardiac myocytes. This pattern correlated (*P *< 0.02, binomial test) with the levels of both hnRNP K and C genes in these tissues (Table S4 in Additional file 3). In total, 46 of 121 (38%) motif pairs in the alternative exon set demonstrated significant tissue-specific gene expression biases (Table S4 in Additional file 3). In almost all cases (18 out of 21) the tissue-specific biases of motif pairs that resemble hnRNP binding sites matched the tissue-specific expression biases of the corresponding hnRNPs (Table S4 in Additional file 3). These data are also in keeping with the idea that the co-occurring motif pairs function in splicing.

### Motif pairs act synergistically to promote splicing

The abundance of co-occurring motif pairs flanking constitutive exons and their absence around pseudo exons, their exclusive residence within 200 nucleotides of the exon borders, their conservation as pairs in evolution, their association with weaker exons and the resemblance of many of them to known RNA binding protein binding sites suggested that the two motifs of a pair may cooperate across an exon to promote exon splicing. To test this idea we used a three-exon minigene with terminal exons and introns derived from the Chinese hamster *dhfr *gene. As a central exon we used exon 2 of the human beta globin gene (Hb2), including the 3' and 5' splice sites, that is, the 223-nucleotide exon plus 14 nucleotides upstream and 6 nucleotides downstream. Deprived of its natural intronic flanks, the Hb2 exon undergoes very little inclusion (approximately 2%) in this context. Tandem pentamers from five co-occurring pairs (numbered 1 to 5) were then inserted in various combinations on either side of the Hb2 exon. Each of the first four pairs (TCCCT/GGAGG, CCCCT/AGGGA, TTTCT/TGGTG, TTTCT/GGTGG) were chosen because they are found in over 1,000 human constitutive exons. The fifth pair (CGCCG/CGCGC) has the most significant *P*-value of association (2.9 × 10^-12^).

**Figure 9:**
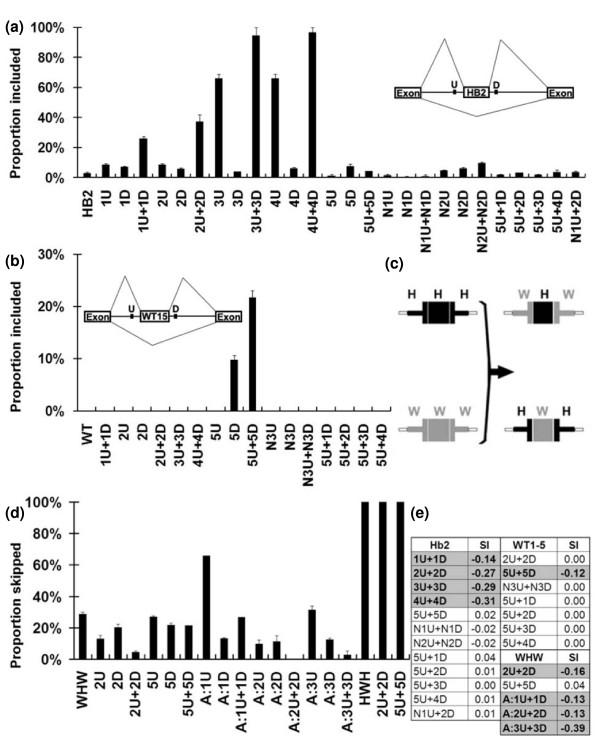
**Co-occurring pairs act synergistically to promote splicing**. **(a) **Splicing of Hb2 exons in transient transfection experiments. The inset diagram is a schematic view of the minigene used. The central exon used to gauge inclusion was exon 2 of the human beta globin gene, including its splice site sequences. Five co-occurring pairs from the UpDp class were tested by inserting two tandem pentamers upstream and/or downstream of the exon. The pairs were: 1, TCCCT_GGAGG; 2, CCCCT_AGGGA; 3, TTTCT_TGGTG; 4, TTTCT_GGTGG; and 5, CGCCG_CGCGC. We also tested two neutral tandem pentamer motifs (N1 and N2) chosen not to resemble any known splicing motifs nor to create such by their insertion nor to create any significantly correlated motif pairs when inserted. U, upstream position; D, downstream position. **(b) **Splicing of WT1-5 exons (exon 5 of the Wilm's tumor gene 1). As (a) but with exon 5 of the Wilm's tumor gene as the central exon, and a different neutral control sequence, N3. **(c) **Diagram of the central exon region of exon body swapped minigenes. **(d) **Splicing of minigene transcripts with exon body swapped exons. Three co-occurring pairs from the UpDp class of alternative exon sets were also tested in this context. The three pairs were: A1, TGGGG:CTGGG; A2, CAGTG:CTTCT; A3, GGGCG:GCGCG. Note that splicing here is measured by percent skipping rather than inclusion. **(e) **Synergy index (SI) of tested pairs. A negative SI signifies synergy, a positive SI signifies anti-synergy and an SI of zero indicates a lack of synergy. Error bars are the standard error of the mean.

Motif pairs 1 to 4 greatly enhanced the inclusion of the Hb2 exon when provided as flanking pairs, yielding 10- to 45-fold increases; motif 5 was without any substantial effect (Figure 9a). When one of the motifs of a pair was omitted, the splicing enhancement was essentially abrogated for six of these eight deletions and was decreased by 30% for the remaining two (3U and 4U; U = upstream, D = downstream; Figure 9a). Combining the two motifs as a pair was synergistic in the sense that the amount of exon inclusion yielded by the pair was always greater than the sum of the enhancements produced by each individual motif. The degree of synergy was verified using the synergy index (see Materials and methods) described by Segre *et al. *[44] and Elena and Lenski [45] (Figure 9e). We also tested two 'neutral' non-co-occurring pairs (N1 and N2) as tandem pentamers. These were carefully chosen to lack resemblance to known splicing motifs [10,19], not to create overlapping sequences of this kind by virtue of their insertion into U and D positions, and not to represent or create motif pairs that exhibited significant correlations as co-occurring pairs. These neutral motifs produced either no or only a small amount of enhancement (Figure 9a). Combining the ineffective motif 5U with any of the effective motifs 1 D to 4 D or combining the effective motif 2 D with the ineffective N1U also failed to promote splicing (Figure 9a). Thus, these pairings exhibited considerable specificity.

To explore the generality of this result, we repeated the experiment using a different central exon, a weakened version of exon 5 of the Wilm's tumor gene (WT1-5: the 51-nucleotide exon body plus 26 nucleotides upstream and 6 nucleotides downstream). Remarkably, the results of co-occurring pair inclusion were now the reciprocal of what was seen for Hb2: co-occurring pairs 1 to 4 were without effect but pair 5 yielded a robust and synergistic 20% exon inclusion (Figure 9b). A third non-co-occurring pair (N3) was again without effect, as were the pairings of the effective 5U with the ineffective (here) 1 D to 4 D motifs. We next asked whether the distinct behavior of the Hb2 and WT1-5 exons was dictated by the exon body sequences or by the different sequences of the splice sites of these two exons. The exon body sequences (from +2 to -4) were swapped between the two exons without changing the 3' and 5' splice sites; these exons are denoted WHW and HWH, where the central letter indicates the exon body sequence and the outside letters the splice sites (Figure 9c). The results are shown in Figure 9d in terms of proportion skipping rather than inclusion. The simple provision of WT-15 splice sites to the Hb2 exon (WHW) greatly promoted splicing, but about 30% exon skipping still took place. Adding co-occurring pair 2 motifs to this exon reduced skipping to only 4%, with the individual motifs being much less effective (Figure 9d). Once again, provision of co-occurring pair 5 motifs was without effect on this chimeric exon. Thus, the effectiveness of the ISE pair was associated with the Hb2 exon body and not its splice sites. Exon HWH failed to splice even when the co-occurring pair 5 was added; apparently the Hb2 3' splice site (splice site sequence consensus value = 72.7 compared to 85.7 for the WT1-5 3' splice site) is too weak to function in this context. The 5' splice site sequence consensus values are strong and comparable at 86.1 for Hb2 and 83.4 for WT1-5.

Since alternative exons by definition often fail to be included, we explored the possibility that co-occurring pairs around alternative exons cooperate to prevent splicing, rather than enhance it. The WHW exon exhibits 70% inclusion in the absence of any added sequences and so provides a sensitive assay for silencing. Three such co-occurring pairs (A1, TGGGG/CTGGG; A2, CAGTG/CTTCT; A3, GGGCG/GCGCG), chosen because they resemble hnRNP binding sites, were inserted in combinations on either side of the WHW exon. All three pairs demonstrated synergy in enhancing exon inclusion, lending no support to the silencing idea (Figure 9d). Interestingly, A1U and A3U silenced exon inclusion as individual motifs, but splicing was re-established when their partners were included (Figure 9d).

These results lead to two conclusions. The first is that the two motifs of a pair are acting not additively but synergistically, suggesting that they act in a concerted manner. The second is that these motifs act in a context-dependent manner, and the specificity of this context can be provided by exon body sequences.

## Conclusions

Exon definition can be thought about at several levels. Perhaps the most fundamental level is as an observable phenomenon: the competence of a splice site at one end of an exon is dependent on competence at the other end [1,2,7,30,46]. At another level it can be thought of as a concept, as a way to impose quality control on splicing; potential exons can be tried out, but if both ends do not pass the test, then the exon is rejected and another is tried. Exon definition can also be considered as a functional complex with biochemically identified components [1,3,4]. In the work described here we found evidence for the coordinated use of ISEs that could play a role in exon definition. A variety of computational evidence supported the conclusion that these elements are functional in splicing and experimental tests validated the idea that pairs of motifs were acting synergistically from the two ends of the exon to promote splicing. These experimental validations also revealed considerable specificity not only in motif pairing but also in interaction with exon body sequences. While it is easy to imagine that interactions between elements spanning an exon are effecting exon definition, exactly what is meant by this process mechanistically remains elusive. Moreover, it may be that these motif pairs are acting at a later step in splicing. For instance, a motif at the 5' end of the downstream intron in a lariat structure may direct interaction with a partner motif just upstream of the exon to hold the cut ends of the RNA together to allow the second step in splicing.

**Figure 10:**
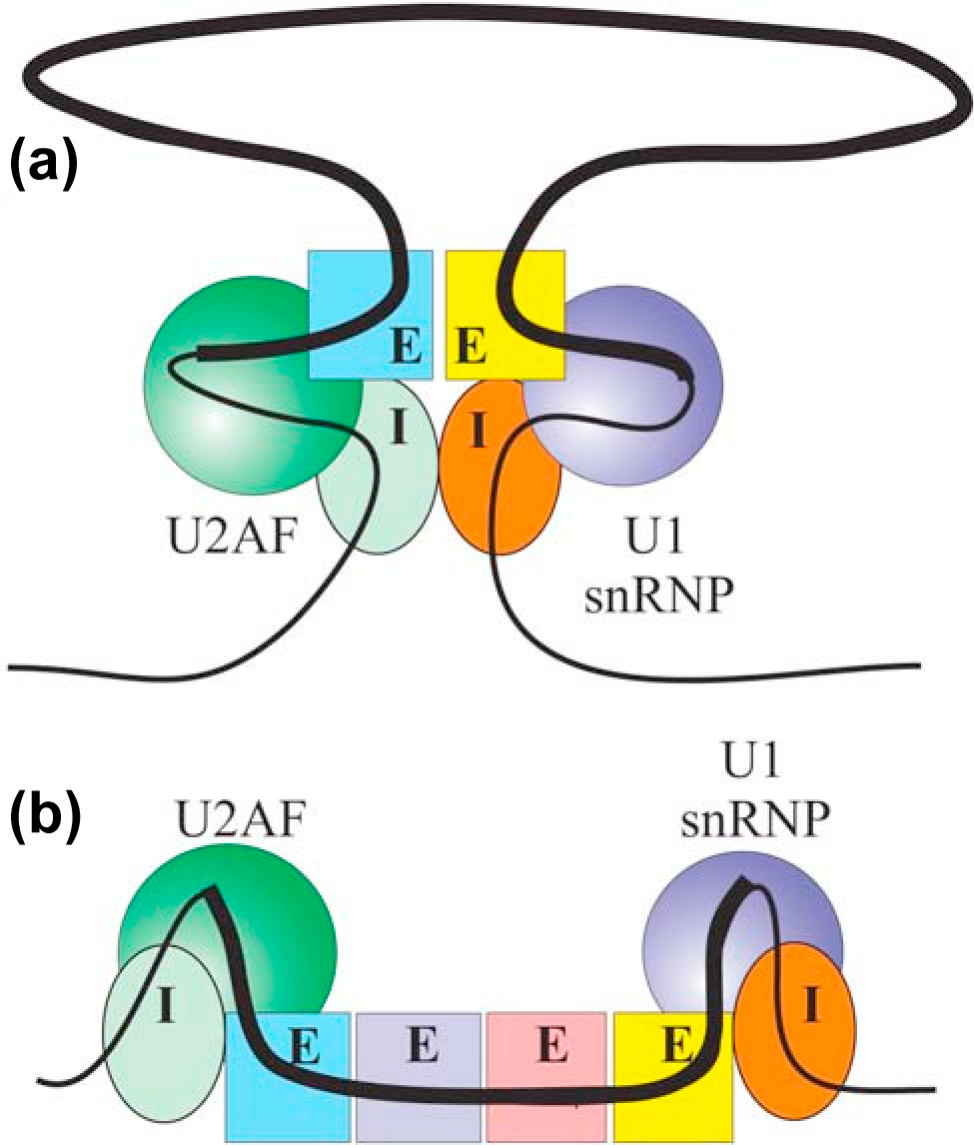
**Models for the role of ISE pairs in exon definition**. **(a) **Proteins binding the co-occurring motif ISE pairs specifically interact with each other as well as factors such as SR proteins binding to two ESEs. This tetrapartite complex can recruit the initial components of the splicing machinery, U1 small nuclear ribonucleoprotein (snRNP) and U2AF, to produce an exon definition complex in which the two ends of the exon are brought together, with much of the exon looped out. It is this complex that can undergo subsequent steps leading to intron definition and splicing. If any one of the four components is missing, the complex is too unstable and efficient recruitment is not realized. **(b) **Proteins binding the co-occurring motif ISE pairs interact specifically with factors such as SR proteins bound to terminally located ESEs. The ESE bound factors interact with additional ESE-binding factors so as to span the entire exon, and it is this exon-wide complex that represents exon definition. The loss of any one component results in destabilization of the complex and loss of exon definition. In either model, some exons may require ISEs and their binding proteins for sufficient stabilization while for others strong ESEs may suffice.

The specificity seen in the tested constructs suggest two exon definition models. In one model (Figure 10a), the two ends of the exon are brought together by a complex of four RNA-binding proteins, two that are bound to co-occurring ISEs and two that are factors bound to ESEs. The interaction with an ESE-binding protein is required in order to account for the exon specificity exhibited by the co-occurring pairs, and two such ESE-binding proteins are proposed to accommodate two different proteins binding to the two different intronic sequences. The interaction of all four of these proteins would be necessary to stabilize the complex for the recruitment of spliceosomal or pre-spliceosomal components, and would explain the specificities of the ISEs for each other and for particular exon body sequences. The second model (Figure 10b) provides another explanation of specificity with a more extensive complex. Here the ISE-binding proteins do not interact with each other but rather with particular terminal components of a network of ESE-binding proteins that spans the exon. Both models imply a requirement for a high order of cooperativity among components with relatively weak individual binding affinities.

## Materials and methods

### Genomic sequences

Human mRNA sequences and ESTs were downloaded from the UniGene database [47] and were aligned to the assembled genomic sequences (hg18) obtained from the human genome sequence [48] using Sim4. Only ESTs that spanned at least two exon-exon joints were considered. Genes that exhibited no intron-exon joints were excluded. Exons with no evidence of skipping or alternative splice site usage were identified as constitutive exons. An exon that was excluded in one or more transcripts and present in at least one transcript was defined as an alternative cassette exon; an exon that demonstrated either alternative 3' or 5' splice site usage in at least one transcript was designated as an alternative 3' or 5' splice site exon. Only exons flanked by canonical AG and GT dinucleotides were included.

For all exons that we used for BBC-COOC analysis, we first removed any repeats defined by RepeatMasker [49]. If two alternative 3' splice sites were present within 50 nts we kept only one (chosen randomly). The same procedure was applied to alternative 5' splice site exons. We further removed highly similar subsets of exons by running BLASTN on the related intronic regions of each exon against those of all other exons in the set. For example, in the UpDp intronic region analysis, if two exons showed similarity in both Up and Dp regions, both exons were purged from the qualified exon sets. Finally, we removed exons having related intronic regions with extreme GC contents of less than 20% or greater than 80%. After application of these three filters we were left with approximately 80,000 constitutive exons and 35,000 alternative exons (alternative cassette, alternative 3' splice site exons and alternative 5' splice site exons) for UpDp, UpDd, UdDp and UdDd analysis.

Pseudo exons were defined as having lengths between 50 and 250 nucleotides and consensus values of ≥75 for 3' splice sites and ≥78 for 5' splice sites. The consensus values were based on a position-specific weight matrix and were calculated essentially according to Shapiro and Senapathy [50]. In addition, pseudo exons had to be at least 100 nucleotides away from the closest real exon. We defined pseudo 3'/authentic 5' splice site exons by searching for a pseudo 3' splice site located at least 100 nucleotides upstream of the authentic 3' splice site of constitutive exons to ensure no overlap with the Ud intronic region. We required the consensus value of a pseudo 3' splice site to be at least as high as that of the authentic 3' splice site of the exon. Authentic 3'/pseudo 5' splice site exons were defined analogously. After removing repeats by RepeatMasker, filtering similar sequences and those with extreme GC contents as above, approximately 100,000 pseudo exons, 18,000 pseudo 3'/authentic 5' splice site exons and 13,000 authentic 3'/pseudo 5' splice site exons were obtained for BBC-COOC analysis.

### BBC-COOC algorithm

The total number of qualified exons for analysis is N. Among these exons we consider intronic pentamer pairs in which one motif occurs upstream and the other downstream of the exon. Of the N exons, n_Ui _exons have a pentamer motif Ui in the upstream intronic region and n_Dj _exons have a pentamer motif Dj in the downstream region. If the existence of motif Ui in the upstream region is independent of the existence of motif Dj in the downstream region, the number of exons with both motif Ui in the upstream region and motif Dj in the downstream region (k) follows the hypergeometric distribution with the probability given by:

$$f(k|N,n_{Ui},n_{Dj})=\frac{\left(\begin{array}{c} n_{Dj}\\ k \end{array}\right)\left(\begin{array}{c} N-n_{Dj}\\ n_{Ui}-k \end{array}\right)}{\left(\begin{array}{c} N\\ n_{Ui} \end{array}\right)}$$

and where the expected value of k is (nUi×nDj)N.

Because the GC contents of the two intronic regions that flank the exon are likely to be highly correlated due to the existence of GC isochores in the human genome, we need to apply a strategy to remove this bias. Toward this end we grouped exons twice, first into 20 rows according the GC content in their upstream regions and then into 20 columns according to the GC content of their downstream regions. The GC content limits were adjusted so that each row contains the same number of exons; the same was done for each column. Thus, each row and each column contain N/20 exons, The result is a grid of 20 × 20 boxes where the exons in each box share a similar GC content both upstream and downstream (Figure S2 in Additional file 1). For motif Ui in a certain row 'y' we enumerated the observed number of exons, M_Ui_, and calculated the frequency of such exons as:

$${\text{q}}_{\text{Ui}}=\frac{M_{Ui}}{N/20}$$

In the same way, for a certain column 'x', we calculated the frequency of exons having motif Dj in a downstream intronic region as:

$${\text{q}}_{\text{Dj}}=\frac{M_{Dj}}{N/20}$$

where M_Dj _is the observed number of exons found to have motif Dj in the downstream intronic region. For the GC box at the intersection of row y and column x, having K_x,y _exons, we estimated the expected number of exons with both motif Ui in the upstream intronic region and motif Dj in the downstream intronic region as k'_x,y _= K_x,y _× q_Ui _× q_Dj_, this expectation being based on the null hypothesis that such coincidence occurs by chance. Based on the linearity of these expected values, we summed the expected number of exons with both motif Ui in the upstream intron and motif Dj in the downstream intron across all GC contents (20 × 20 boxes). The final sum is:

$$k'=\sum_{x=1}^{20}\sum_{y=1}^{20}k{'}_{x,y}$$

which is the expected total number of exons with both motif Ui in the upstream intron and motif Dj in the downstream intron according to the null hypothesis. This carefully calculated expectation takes into account the GC content correlation between upstream and downstream intronic regions of an exon. Of all the N exons, n_Ui _exons have motif Ui in the upstream intron and n_Dj _exons have motif Dj in the downstream intron. We need to adjust n_Ui _or n_Dj _or both to make k' equal the theoretical mean of the hypergeometric distribution (nUi×nDj)N. We kept n_Ui _the same and adjusted n_Dj _to:

nDj'=N×k'nUi

Adjusting n_Ui _or adjusting both yielded similar results. It is this adjustment that takes the GC content correlation into account. We next counted the actual number of exons with both motif Ui in the upstream intron and motif Dj in the downstream intron (k^a^) across all 20 × 20 boxes:

$$k^{a}=\sum_{x=1}^{20}\sum_{y=1}^{20}k_{x,y}^{a}$$

Under a null hypothesis, we would expect k^a ^to follow the same hypergeometric distribution as k' with a probability:

$$f(k^{a}|N,n_{Ui},n_{Dj}^{'})=\frac{\left(\begin{array}{c} n_{Dj}^{'}\\ k^{a} \end{array}\right)\left(\begin{array}{c} N-n_{Dj}^{'}\\ n_{Ui}-k^{a} \end{array}\right)}{\left(\begin{array}{c} N\\ n_{Ui} \end{array}\right)}$$

From this distribution we can calculate the *P*-values for all observed values of k^a^, where k^a ^values greater than the mean indicate a positive correlation and *vice versa*. This method of dealing with the biased GC contents is similar to one used by the Burge laboratory to discover co-occurring pairs across introns [15] except for our use of the hypergeometric distribution, rather than a Poisson approximation.

### Co-conservation of co-occurring motif pairing

Based on the coordinates of the human exons, orthologous macaque exons were extracted from a 17-genome multi-alignment [51]. In this way were able to survey a total of 59,221 constitutive exon pairs. We then identified human exons that had co-occurring pairs in their related intronic regions. We considered a human intronic motif to be conserved if it also existed anywhere within the related intronic region of the macaque orthologous exon (that is, performing no sequence alignment). We defined conservation of upstream pairing as the proportion of those exons with a conserved downstream motif that have also conserved the upstream partner. Conservation of downstream pairing was similarly defined. As a control, for each regional class we used two groups of paired motifs that are non-co-occurring. Non-co-occurring here signifies a motif that has the same GC content as the co-occurring motif, but that generates a *P*-value of >0.05 with its original partner. For example, for the non-co-occurring motif pair UpDp_non-pairing_, Dp_non-pairing _had the same GC content as the corresponding Dp but formed a non-co-occurring pair with Up; and for Up_non-pairing_Dp, Up_non-pairing _had the same GC content as the corresponding Up but formed a non-co-occurring pair with Dp.

Human SNP data were downloaded from dbSNP build 130 [52]. SNPs that mapped to multiple genomic regions or known repetitive elements were excluded. The density of SNPs was calculated separately for each class of motifs when co-occurring and when alone.

### Tissue expression bias analysis

From BioGPS [53] we obtained the Affymetrix HG-133A and GNF1B microarray gene expression data from 79 human tissues and cell lines [43]. Mappings for Affymetrix probe identifiers were downloaded from [54]. Genes expressed in a given tissue or cell line at greater than two standard deviations above the median expression across all 79 tissues and cell lines were defined as tissue-specifically expressed. For each co-occurring motif pair, we identified all exons that have the pair in the related intronic regions, and the corresponding genes that contain these exons. For each tissue/cell line, the fraction of genes showing tissue-specific expression, F_E,Tissue_, was calculated. As a control, we collected the same number of randomly chosen exons that did not contain the co-occurring pair (10,000 iterations). The number of exons in each control gene was matched to the same number of exons in the genes with co-occurring motifs. The fraction of control genes associated with each tissue/cell line was then calculated, F_C,Tissue_. The *P*-value for the significance of enrichment (or depletion) for each tissue/cell line was computed for the fraction of times that F_E,Tissue _was lower than or equal to F_C,Tissue _(F_E,Tissue _was higher than or equal to F_C,Tissue _for depletion). The *P*-value cutoff for significance of tissue/cell lines bias was 0.001.

### Experimental validation of co-occurring motifs

To examine the cooperative activity of co-occurring pairs in splicing, we used a hamster *dhfr *minigene modified from that previously described [16]. The starting minigene contains a central merged exon 2-3 flanked by exon 1 and a merged exon 4-6. This mini-gene is driven by a cytomegalovirus (CMV) promoter and is terminated by the SV40 late poly A site. Around the middle exon, a NotI site has been inserted in intron 1 and a natural NheI site occurs in the second intron. We tested two exons as the middle exon: human beta globin gene exon 2 (Hb2), and the Wilms tumor gene 1 exon 5 (WT1-5) with two point mutations in its exon body. Hb2 and WT1-5 were amplified without their flanking intronic sequences beyond the splice site consensus sequences (upstream 14 nucleotides and downstream 6 nucleotides for Hb2, upstream 26 nucleotides and downstream 6 nucleotides for WT1-5) using primers tailed with NotI or NheI restriction sites. The amplified fragments were cut by NotI and NheI and inserted as the middle exon of the mini-gene construct. Pentamers U and D of a pair were inserted as tandem duplicates between the NotI site and the 3' splice site region and between 5' splice site region and the NheI site of the tested exons using synthetic double-stranded oligomers. Neutral non-co-occurring pentamer pairs were chosen so as neither to form co-occurring pairs nor to create any known splicing motifs after insertion (that is, also considering the overlaps formed). We used the merged hexamer set (hexESEs and hexESSs) from our previous study [10] and the ISRE list by Yeo and colleagues [19] as the known splicing motifs to avoid.

HEK 293 cells cultured in 35 mm dishes were transfected using Lipofectamine 2000 (Invitrogen, Carlsbad, CA, USA) following the manufacturer's protocol. After cells were incubated for 24 hours, total RNA was extracted using illustra RNAspin Mini Kits (GE Healthcare, Piscataway, NJ, USA). A sample of 400 ng of RNA was reverse transcribed (RT) using Omniscript (Qiagen, Valencia, CA, USA) and a specific primer, AGAGTCTGAGATGGCCTGGCT, which pairs with a region in the third exon. One-tenth of the RT product was used as template in the following PCR amplification: forward primer, GTCAGATCCGCCTCCGCGTA; reverse primer, GTAAACGGAACTGCCTCCAA; initial denaturation, 94°C for 2 minutes; denaturation, 94°C for 45 s; annealing, 60°C, 1 minute; extension, 72°C, 1 minute; 20 cycles; final extension, 72°C, 5 minutes. Splicing products were separated in 1.8% agarose gels stained with ethidium bromide; the intensity of each splicing product was quantified with ImageJ. At least two independent transfections were performed for each construct. Proportion included (I) was defined as Included product/(Skipped product + Included product) in molar quantities.

### Definition of synergy index (SI)

By analogy to previous synergy calculations [44,45], we focused on the effect of our experimental perturbations on exon skipping. Proportion skipped (S) was defined as Skipped product/(Skipped product + Included product). For the insertion of motif U, skipping 'fitness' was defined as W_U _= S_U_/S_no-insertion_. For motif pair U:D, we evaluated the level of synergy by comparing the skipping fitness W_UD _of the pair insertion construct with the product of the skipping fitness values W_U _and W_D _of the corresponding single motif insertion constructs. The synergy index for promoting splicing inclusion is defined as SI = W_UD _- W_U _× W_D_. SI <0 indicates synergy, SI = 0 indicates no synergy, and SI >0 indicates anti-synergy [44,45].

## Abbreviations

BBC-COOC: base bias corrected co-occurrence; Dd: downstream distal; Dp: downstream proximal; ESE: exonic splicing enhancer; ESS: exonic splicing silencer; EST: expressed sequence tag; Hb2: human beta globin gene; hnRNP: heterogeneous nuclear ribonucleoprotein; ISE: intronic splicing enhancer; SI: synergy index; SNP: single-nucleotide polymorphism; Ud: upstream distal; Up: upstream proximal; WT1-5: exon 5 of the Wilm's tumor gene 1.

## Authors' contributions

SK and LC conceived and planned the research, analyzed the data and wrote the manuscript. SK carried out the experiments.

## Supplementary Material

Additional file 1 **Figures S1, S2, and S3, and Tables S1 and S3**. Figure S1: distribution of pentamer pairs around constitutive exons. Figure S2: scheme for grouping exons according to the GC content of their flanks. Figure S3: tissue-specific expression of six hnRNPs. Table S1: results with no GC balancing. Table S3: overlap of UpDp downstream motifs with other reported intronic motif sets. Click here for file

Additional file 2 **Table S2**. List of co-occurring pairs. Click here for file

Additional file 3 **Table S4**. Tissue-specific expression of genes with co-occurring motif pairs. Click here for file
