# Supplementary material for: Intronic motif pairs cooperate across exons to promote pre-mRNA splicing
Source: Genome Biol. 2010 Aug 12;11(8):R84. doi: 10.1186/gb-2010-11-8-r84 (PMC2945786; doi:10.1186/gb-2010-11-8-r84)
Supplement: Additional file 1 — Figures S1, S2, and S3, and Tables S1 and S3. Figure S1: distribution of pentamer pairs around constitutive exons. Figure S2: scheme for grouping exons according to the GC content of their flanks. Figure S3: tissue-specific expression of six hnRNPs. Table S1: results with no GC balancing. Table S3: overlap of UpDp downstream motifs with other reported intronic motif sets. [file gb-2010-11-8-r84-S1.PDF]

Ke and Chasin: Additional figures and tables

Supplementary material for:
Intronic motif pairs cooperate across exons to promote pre-mRNA splicing

Shengdong Ke and Lawrence A. Chasin[‡]
Department of Biological Sciences
Columbia University
New York, NY 10027 USA

[‡] To whom correspondence should be addressed:

Dept. of Biological Sciences, Columbia University
1212 Amsterdam Ave., MC 2433
New York, NY 10027 USA
212-854-4645
Fax: 212-531-0425
Email: lac2@columbia.edu

Additional Figures :
Figure S1. Distribution of pentamer pairs around constitutive exons
Figure S2. Scheme for grouping exons according to the GC content of their flanks
Figure S3. Tissue specific expression of six hnRNPs

Additional Tables:
Table S1. Results with no GC balancing
Table S2. List of co-occurring pairs: *see link to separate file in paper*.
Table S3. Results with no GC balancing
Table S4. Tissue specific expression of genes with co-occurring motif pairs: *see link to separate file in paper*

Figure S1. Distribution of pentamer pairs around constitutive exons. a) With no GC balancing, the top motif pairs detected exclusively shared similar GC content, being either GC-rich or AT-rich. b) The GA content correlations of the UpDp intron region pairing is close to zero (r = -0.01). c) The GT content correlations of the UpDp intron region pairing is close to zero (r = 0.04).

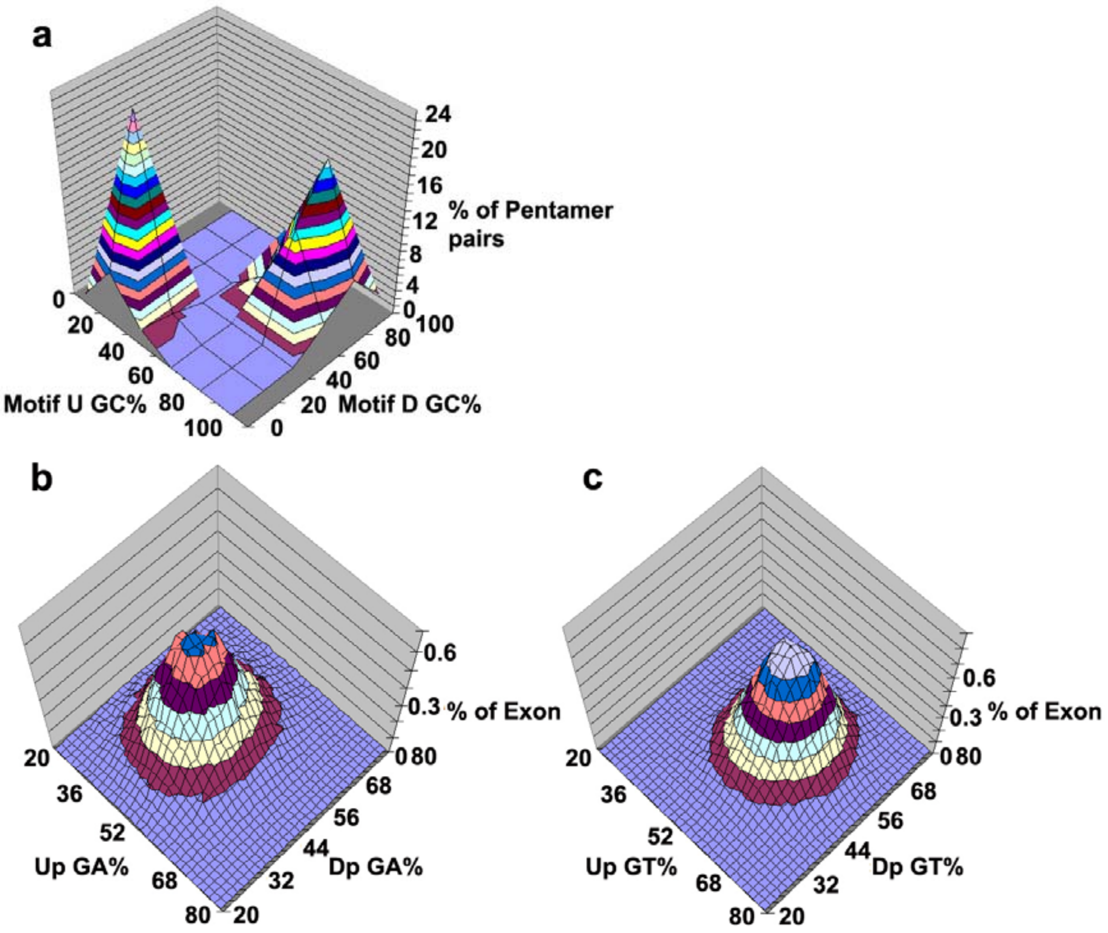

Figure S2. Scheme for grouping exons according to the GC content of their flanks. All exons were divided equally among 20 different rows; exons in each row share a similar upstream intronic GC content. All exons were also divided equally into another 20 groups based on their downstream intronic GC content. Each row and column intersect to form a box in which all the exons share a similar GC content in both upstream and downstream intronic regions.

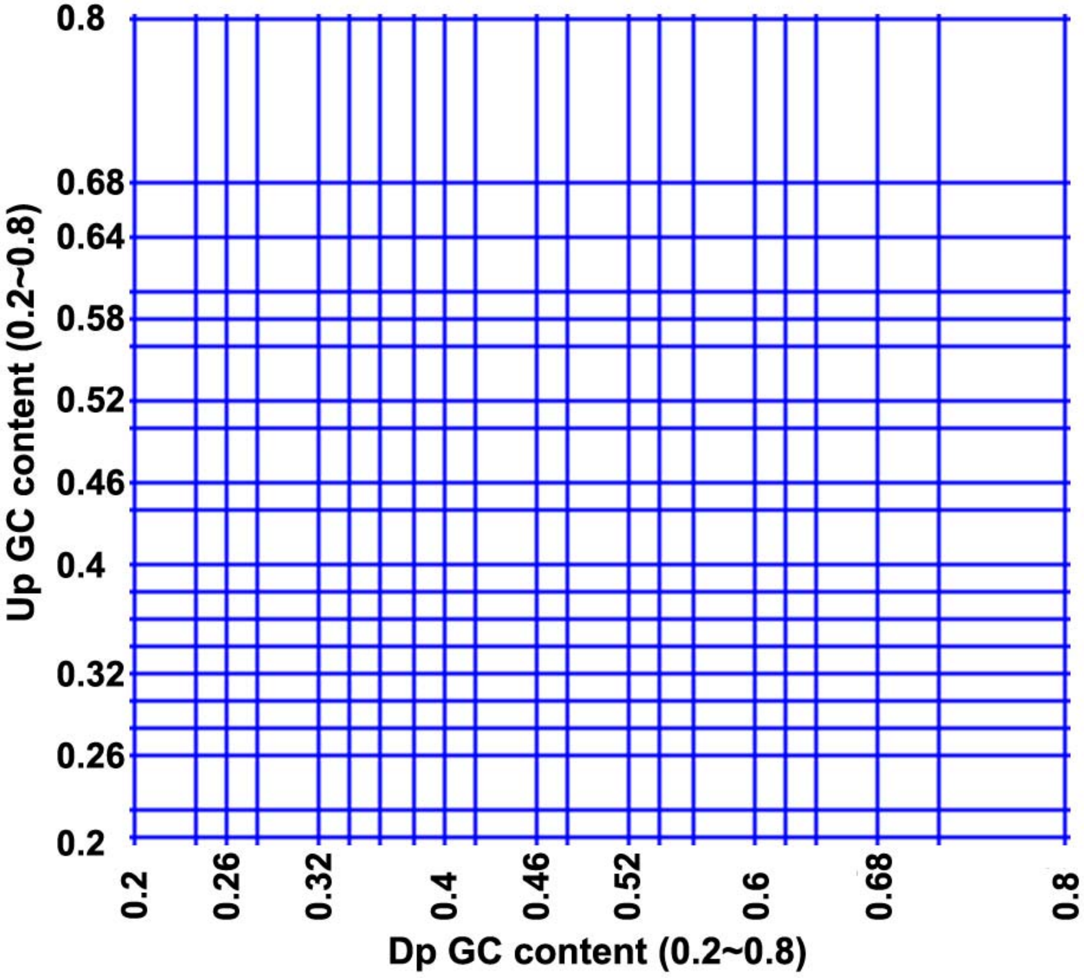

Figure S3. Tissue specific expression of six hnRNPs. Relative expression was calculated from microarray data of Su et al. *(Proc Natl Acad Sci U S A* **101**, 6062-7, 2004). Each individual value was divided by the median value across all 79 tissues and cell lines examined and the log base 2 of that number is indicated on the ordinate. Thus expression values greater than the median are positive and those less than median are negative.

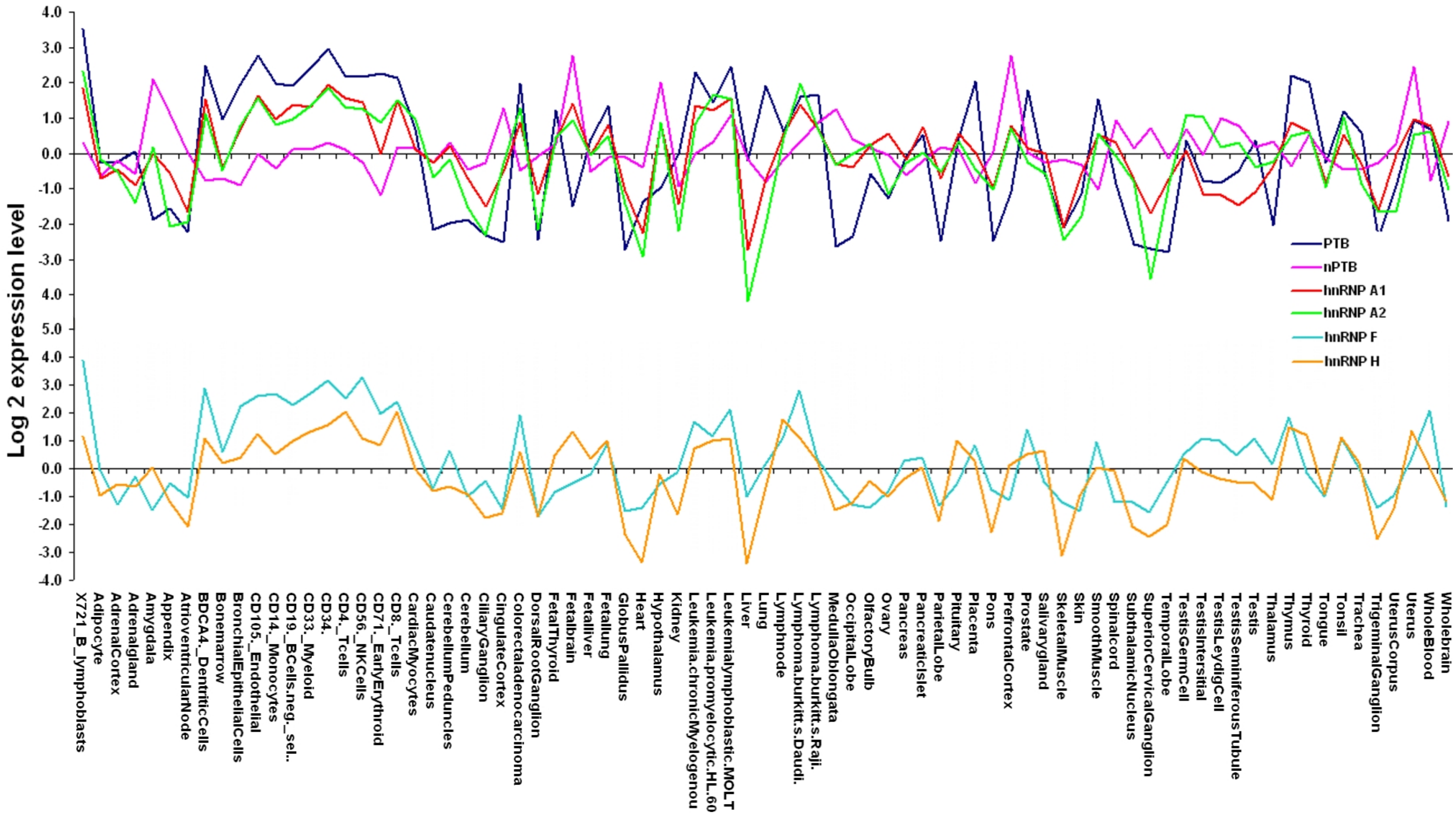

0.0134

0.0000

Table S1. Results with no GC balancing. With no GC balancing, the top motif pairs detected in this analysis exclusively shared similar GC content, being either GC-rich or AT-rich. Each number represents the proportion of motif pairs having the indicated GC contents. Only the UpDp analysis is shown, all others being similar.

		Proportion Up GC content					
		0	0.20.0000	0.4	0.6	0.8	1
Proportion Dp GC content	0	0.0135	0.0670	0.0210	0.0000	0.0000	0.0000
	0.2	0.0668	0.2322	0.0127	0.0000	0.0000	0.0000
	0.4	0.0085	0.0059	0.0001	0.0000	0.0000	0.0000
	0.6	0.0000	0.0000	0.0000	0.0346	0.1213	0.0273
	0.8	0.0000	0.0000	0.0000	0.0785	0.1784	0.0568
	1						

Table S3. Results with no GC balancing

Motif set	Selection	5-mers	Exp.	Obs.	Reference
Zhang et al. 2005 ISEs	Statistical + conservation	43	1.8	4	Genome Res **15**, 768-79 (2005)
Yeo ISR 5-mers	Conservation	103	3.5	1	PLoS Genet **3**, e85 (2007)
Voelker ISR 5-mers	Conservation	153	5.2	5	Genome Res **17**, 1023-33 (2007)
Zhang 2003 ISSs	Machine learning	68	2.5	0	*Genome Res* **13**, 2637-50 (2003).
Yu ISSs 5-mers	Biochemical selection	194	7.2	2	*Cell* **135**, 1224-36 (2008)

[a] The 38 unique downstream pentamers of the UpDp sets for constitutive and alternative exons were used as a basis for comparison. When necessary 5-mers were extracted from longer oligomers in a set.
